# PPARγ Pro12Ala and ACE ID polymorphisms are associated with BMI and fat distribution, but not metabolic syndrome

**DOI:** 10.1186/1475-2840-10-112

**Published:** 2011-12-14

**Authors:** Angela Passaro, Edoardo Dalla Nora, Caterina Marcello, Francesca Di Vece, Mario Luca Morieri, Juana M Sanz, Cristina Bosi, Renato Fellin, Giovanni Zuliani

**Affiliations:** 1 Department of Clinical and Experimental Medicine, Section of Internal Medicine, Gerontology and Clinical Nutrition, University of Ferrara, Ferrara, Italy

**Keywords:** PPARγ2, Pro12Ala, ACE I/D, polymorphism, Metabolic Syndrome, obesity, fat distribution

## Abstract

**Background:**

Metabolic Syndrome (MetS) results from the combined effect of environmental and genetic factors. We investigated the possible association of peroxisome proliferator-activated receptor-γ2 (PPARγ2) Pro12Ala and Angiotensin Converting Enzyme (ACE) I/D polymorphisms with MetS and interaction between these genetic variants.

**Methods:**

Three hundred sixty four unrelated Caucasian subjects were enrolled. Waist circumference, blood pressure, and body mass index (BMI) were recorded. Body composition was estimated by impedance analysis; MetS was diagnosed by the NCEP-ATPIII criteria. A fasting blood sample was obtained for glucose, insulin, lipid profile determination, and DNA isolation for genotyping.

**Results:**

The prevalence of MetS did not differ across PPARγ2 or ACE polymorphisms. Carriers of PPARγ2 Ala allele had higher BMI and fat-mass but lower systolic blood pressure compared with Pro/Pro homozygotes. A significant PPARγ2 gene-gender interaction was observed in the modulation of BMI, fat mass, and blood pressure, with significant associations found in women only. A PPARγ2-ACE risk genotype combination for BMI and fat mass was found, with ACE DD/PPARγ2 Ala subjects having a higher BMI (p = 0.002) and Fat Mass (p = 0.002). Pro12Ala was independently associated with waist circumference independent of BMI and gender.

**Conclusions:**

Carriers of PPARγ2 Ala allele had higher BMI and fat-mass but not a worse metabolic profile, possibly because of a more favorable adipose tissue distribution. A gene interaction exists between Pro12Ala and ACE I/D on BMI and fat mass. Further studies are needed to assess the contribution of Pro12Ala polymorphism in adiposity distribution.

## Background

Epidemiological studies have demonstrated that glucose intolerance, hypertension, and abdominal obesity often coexist in the same individuals increasing the risk for coronary heath disease (CHD). In 1988, Reaven focused on this cluster of cardiovascular risk factors, named it as "syndrome X", and proposed insulin resistance as the possible common etiological factor [1]. The relationship between insulin resistance and metabolic risk factors is not fully understood and appears complex. Most persons with insulin resistance have abdominal obesity, and dysfunctional adipose tissue might be the common link in determining this cluster of risk factors, currently named as metabolic syndrome (MetS).

Among the clinical criteria proposed for the diagnosis of MetS, the one provided by the National Cholesterol Education Program-Adult Treatment Panel III (NCEP-ATPIII) is easily applicable, and identifies patients with different combinations of hypertension, atherogenic dyslipidemia, impaired glucose homeostasis, and visceral obesity [2]. It is difficult to dissect the possible contribution of each MetS component to CHD risk; however, there is little doubt that in individuals with MetS the CHD risk is increased, and that insulin resistance enhances the risk of developing type 2 diabetes.

The pathogenesis of MetS has been associated with the effect of a genetic predisposition [3] in combination with environmental factors. Considering the central role of adipose tissue in MetS, different adipocyte related genes have been studied as possible candidates in MetS, including peroxisome proliferator-activated receptor-γ (PPARγ), and renin-angiotensin system related genes. PPARγ transcriptionally regulates the expression of genes involved in cell metabolism; endogenous ligands are thought to bind PPARγ and promote downstream gene target transcription [4,5]. From human PPARγ gene two distinct isoforms of mRNA and protein, PPARγ1 and PPARγ2, are spliced [6]. PPARγ 1 is abundantly expressed in different tissues including adipose tissue and macrophages [7], whereas PPARγ 2 expression is restricted only to adipose tissue. In vivo and in vitro studies demonstrated that PPARγ has a critical role in regulating adipocyte differentiation and lipid accumulation [8,9]; furthermore, its has been implicated in the regulation of lipid homeostasis and insulin sensitivity [10]. Not surprisingly, PPARγ has been identified as the target for thiazolidinediones [11], drugs that improve insulin sensitivity [10]. The role of PPARγ in affecting insulin action has been confirmed by several genetic studies on PPARγ gene polymorphisms; among these, the Pro12Ala mutation in PPARγ 2 (Pro12Ala) is the most common [12]. The Ala12 variant has been associated with decreased body mass index (BMI) [13-16], increased BMI [17,18], and increased risk of obesity [19]. Different meta-analysis on Pro12Ala mutation and type 2 diabetes indicates that Ala12 carriers have a reduced risk of diabetes compared to Pro12 carriers [20,21]; nevertheless, the association between Pro12Ala and type 2 diabetes seems to be modulated by genetic and environmental factors, dietary lipids [22], and intrauterine condition [23].

Recently, the Angiotensin Converting Enzyme (ACE) gene also received substantial attention as possible candidate for diabetes [24], hypertension [25], cardiovascular disease [26], and diabetic nephropathy [27]. Recent data suggest that the Renin Angiotensin System might be involved in the pathophysiology of obesity and associated hypertension [28]; therefore, ACE gene might be a good candidate for MetS. The most common ACE gene polymorphism is the insertion-deletion (I/D) located in intron 16; the D allele has been associated with higher levels of circulating ACE [29,30]. Moreover PPARs modulate the renin-angiotensin-aldosterone system (RAAS), by transcriptional control of renin, angiotensinogen, angiotensin converting enzyme (ACE) and angiotensin II receptor 1 (AT-R1) [31], linking biologically the Renin Angiotensin System with the PPARs. Despite data on the association between ACE gene and MetS or hypertension are not conclusive, it appears interesting to evaluate the possible interaction of these two common polymorphisms.

In the present study we investigated, in a cohort of Italian adult individuals, the possible association between PPARγ2 Pro12Ala and ACE I/D polymorphisms and MetS components; furthermore, we searched for a possible interaction between these two common genetic variants.

## Methods

### Subjects

From January 2007 to December 2008, 364 outpatients consecutively referred to our clinic for metabolic disorders, at the S. Anna University Hospital (Ferrara, Italy), were enrolled. All patients were unrelated and Caucasian. They underwent a complete medical interview and medical examination; arterial blood pressure was registered. 27.5% of subjects were taking antidiabetic drugs while 39% were taking antihypertensive therapy. Their BMI was calculated (Kg/m^2^), and waist circumference was measured between the lower rib and the iliac crest at the end of a normal expiration. Body composition was estimated by impedance analysis (Human IM plus II^®^, DS Medica, Milan, IT). After overnight fasting, a blood sample was drawn for biochemical analysis and DNA extraction.

MetS components were diagnosed in accordance with the NCEP-ATPIII criteria (2), assigning each subject an individual score from 0 (no MetS features) to 5 (five MetS features). Subjects having a score ≥3 were considered as affected by MetS. The Study was approved by the local ethic committee and all participants gave their written consent.

### Biochemical analysis

Fasting glucose was assessed by glucose oxidase method (Glucose Analyzer II, Beckmann Instruments, Fullerton, CA, USA), while plasma total cholesterol and triglycerides were evaluated by standard enzymatic techniques (Boehringer Mannheim-Diagnostica, Mannheim, Germany). Low-density lipoprotein (LDL)-cholesterol was calculated by the Friedewald formula. Serum concentrations of high-density lipoprotein (HDL) cholesterol were measured after precipitation of apoprotein B with phosphotungstic acid-MgCl2 and by homogeneous method [10]. Fasting insulin was measured by standard chemiluminescent assay (Access System, Beckman, Palo Alto, CA, USA).

### Genotyping

Genomic DNA was extracted from peripheral blood using standard technique [32]. The Pro12Ala PPARγ 2 variant (rs1801282) was detected by polymerase chain reaction-restriction fragment length polymorphism (PCR-RFLP) analysis. The analysis, as previously described [12], uses a mutagenic PCR primer to introduce a BstU I restriction site only when a C →G substitution at nucleotide 34 of the PPARγ 2 gene is present. Genotyping was repeated in all Ala12 homozygotes, all Pro12Ala heterozygotes, and in randomly selected Pro12 homozygotes; reproducibility was 100%.

A DNA fragment on intron 16 of the ACE gene was amplified by PCR to determine the ACE I/D genotype (rs4340). The sequences of flanking primers used were 5'-CTGGAGACCACTCCCATCCTTTCT-3' for the sense primer and 5'-GATGTGGCCATCA CATTCGTCAGAT-3' for the antisense primer. PCR amplification products were obtained as previously described [33]. To avoid ID-DD mistyping, a pair of primers that amplify a region inside intron 16 was also used to analyze all samples showing DD genotype [33].

### Statistical analysis

Because of the low number of Ala/Ala homogygotes (n = 5), Pro/Ala and Ala/Ala carriers were pooled together in statistical analysis (named XA). Variables with normal distribution are expressed as mean ± standard deviation while variables with a not normal distribution are expressed as median and interquartile range and were log transformed before entering the statistical analysis. Means were compared by ANOVA using the Bonferroni Post Hoc Tests for post-hoc analysis, while medians were compared by non-parametric tests (Kruskal Wallis test). Correlations between continuous variables were tested by multivariate linear regression analysis. The prevalence of MetS among different genotypes was tested by the Chi-square test.

To test the hypothesis that the relationship between BMI and waist circumference might be different according to PPARγ2 genotype, we performed a 2 ways ANOVA comparing slopes of the two regression lines.

The possible association between waist circumference and other variables of interest (BMI, gender and PPARγ2polymorphism) was tested by multivariate linear regression analysis.

Interaction between gene polymorphisms in modulating BMI and fat mass was tested by, age weighted, general linear model univariate analysis.

All statistical tests were performed using SPSS 16.0 software (SPSS Inc., Chicago, IL).

## Results

The principal clinical features of the whole sample across different PPARγ2 and ACE I/D genotypes are reported in Table 1. We found 5 Ala/Ala subjects (1.4%), 25 Pro/Ala subjects (7%) and 334 Pro/Pro subjects (92%). Ala allele frequency appears to be consistent with previous reports [34].

**Table 1:** Clinical features of the whole study population across different PPARγ2 and ACE I/D genotypes

			All subjects				
		
	Pro12Ala			ACE I/D			
		
	PP (334)	XA (30)	p	DD (137)	ID (172)	II (55)	p
Age (years)	56 ± 13	53 ± 10	0.200	56 ± 12	55 ± 14	56 ± 13	0.805
**BMI (kg/m2)**	***30.6 ± 5.7***	***33.2 ± 6.3***	***0.015***	31.3 ± 6.3	30.4 ± 5.4	30.9 ± 5.5	0.402
WAIST (cm)	98.4 ± 13.9	100.1 ± 13.8	0.528	99.8 ± 14.5	97.5 ± 13.2	98.7 ± 14.2	0.360
**Fat Mass (kg)**	***30.1 ± 11.9***	***35.7 ± 13.1***	***0.016***	31.1 ± 12.8	29.9 ± 11.6	31.1 ± 12.2	0.644
Glucose (mmol/L)	6.5 ± 2.6	5.9 ± 2.3	0.271	6.7 ± 2.9	6.2 ± 2.3	6.5 ± 2.5	0.22
Insulin (μUI/L)*	7.8 (5.1-12.2)	10.1 (8.0-15.6)	0.284	8.0 (6.0-11.7)	7.9 (5.0-13.0)	8.9 (5.3-14.5)	0.338
HOMA Insulin Resistance*	2.1 (1.3-3.4)	2.3 (1.9-3.6)	0.668	2.1 (1.5-3.3)	2.1 (1.2-3.5)	2.4 (1.3 -3.9)	0.816
**Systolic Blood Pressure (mmHg)**	***134.1 ± 17.4***	***126.8 ± 17.2***	***0.033***	132.3***± ***17.1	134.7***± ***18.1	132.6***± ***16.3	0.486
Diastolic Blood Pressure (mmHg)	82.4 ± 8.5	81.6 ± 10.6	0.637	82.4 ± 9.2	82.6 ± 8.3	81.2 ± 8.5	0.612
Total Cholesterol (mmol/L)	5.6 ± 1.4	5.9 ± 1.1	0.245	5.8 ± 1.4	5.7 ± 1,4	5.3 ± 1,2	0.1
Triglyceride (mmol/L)*	1.46 (1.04-2.07)	1.32 (0.99-1.93)	0.566	1.54 (1.13-2.15)	1.4 (0.99-2.01)	1.43 (0.97-2.0)	0.41
HDL cholesterol (mmol/L)	1.3 ± 0.4	1.4 ± 0.3	0.595	1.3 ± 0.4	1.3 ± 0.4	1.4 ± 0.3	0.92
LDL Cholesterol (mmol/L)	3.5 ± 1.3	3.8 ± 1.1	0.199	3.6 ± 1.2	3.6 ± 1.3	3.2 ± 1.1	0.16

*Median (Interquartile range)

Compared with Pro/Pro homozygotes (PP), PPARγ2 Ala carriers were characterized by higher BMI (p: 0.015) and higher fat-mass (p:0.016), but lower systolic blood pressure (p:0.033). No significant differences in waist circumference and other metabolic features were observed. Subjects carrying different ACE polymorphisms did not differ in terms of BMI, waist circumference, blood pressure, fasting glucose-insulin levels, and lipid profile.

A significant PPARγ2 gene-gender interaction was observed in the modulation of BMI, fat-mass and systolic blood pressure (Table 2). Indeed, when analysis was separately conducted by gender, XA women (but not men) showed higher BMI (p: 0.029) and fat-mass (p: 0.028) compared with PP. Similarly, a trend toward lower values of systolic blood pressure was observed in XA women (but not men) compared with PP (p: 0.07).

**Table 2:** Gender based dimorphism of PPARγ2 gene polymorphism on clinical features of the study population

	PPARγ2 Pro12Ala					
		
	Men			Women		
		
	PP (129)	XA (8)	p	PP (205)	XA (22)	p
Age (years)	57 ± 14	57 ± 14	0.970	55 ± 13	51 ± 8	0.141
**BMI (kg/m2)**	29.98 ± 4.75	31.20 ± 6.69	0.492	***30.98 ± 6.19***	***34.02 ± 6.16***	***0.029***
WAIST (cm)	102.40 ± 12.76	104.19 ± 15.04	0.705	95.92 ± 14.05	98.61 ± 13.38	0.392
**Fat Mass (kg)**	27.35 ± 10.62	29.16 ± 13.56	0.646	***31.88 ± 12.47***	***38.08 ± 12.42***	***0.028***
Glucose (mmol/L)	7.1 ± 2.8	5.9 ± 2.1	0.278	6.1 ± 2.4	5.9 ± 2.4	0.737
Insulin (μUI/L)*	7.7 (5.2-12.0)	7.2 (4.0-14.4)	0.778	7.9 (5.1-12.6)	10.08 (8.6-15-7)	0.210
HOMA Insulin Resistance*	2.4 (1.5-3.5)	1.9 (0.9-3.6)	0.466	2.0 (1.3-3.3)	2.4 (2.1-3.6)	0.416
**Systolic Blood Pressure (mmHg)**	136.93 ± 15.09	134.17 ± 6.65	0.657	***132.38 ± 18.51***	***124.77 ± 18.67***	***0.070***
Diastolic Blood Pressure (mmHg)	82.57 ± 8.23	84.17 ± 7.36	0.642	82.33 ± 8.69	80.91 ± 11.41	0.485
Total Cholesterol (mmol/L)	5.5 ± 1.5	5.5 ± 1.0	0.939	5.7 ± 1.3	6.1 ± 1.1	0.206
Triglyceride (mmol/L)*	1.76 (1.12-2.5)	1.3(1.05-1.9)	0.414	1.37 (0.96-1.79)	1.32 (0.96-1.93)	0.687
HDL cholesterol (mmol/L)	1.2 ± 0.3	1.4 ± 0.3	0.155	1.4 ± 0.3	1.4 ± 0.4	0.463
LDL Cholesterol (mmol/L)	3.4 ± 1.4	3.4 ± 0.9	0.918	3.6 ± 1.2	4.0 ± 1.1	0.162

*Median (interquartile range)

**Figure 1:**
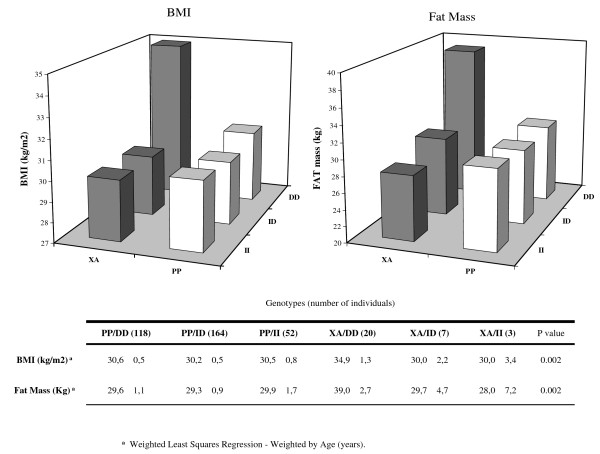
**Interaction analysis between PPARγ2 Pro12Ala and ACE I/D polymorphisms on BMI and fat mass**. In the graph BMI (left chart) and Fat Mass (right chart) were plotted according to all 6 different genotype combination. In the lower panel Mean ± SD and p value for univariate regression analysis, age weighted, are reported.

The combined effect of PPARγ-ACE genotypes on BMI, fat-mass and waist circumference is shown in Figure 1. In individuals homozygous for ACE D allele, those bearing a PPARγ Ala allele (XA/DD) showed higher BMI (p:0.002) and higher fat-mass (p:0.002).

**Figure 2:**
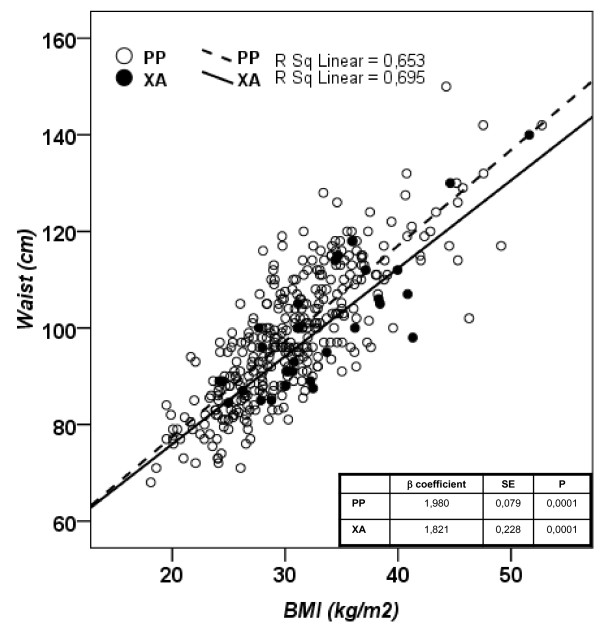
**Linear regression between BMI and waist circumference according to different PPARγ2 Pro12 Ala genotype (subjects bearing Ala allele: black circles; subjects Pro homozygotes: white circles)**.

Figure 2 reports the two linear regressions for BMI vs waist circumference according to PPARg2 Pro12Ala genotypes (PP and XA). BMI correlated with waist circumference in both group; however, the slope tend to be lower in XA compared with PP individuals (Slope XA 1,817 ± 0,2275, slope PP 1,975 ± 0,07910, p:0.53).

By multivariate linear regression analysis we were able to demonstrate that the PPARγ2 Pro12Ala polymorphism (as categorical variable: PP:0; XA:1) was significantly associated with waist circumference (β coefficient: -2.73; p: 0.04) independent of BMI (β coefficient: 2.03; p: 0.001), and gender (β coefficient: 8.75; p: 0.001) (p for model: 0.001).

The number of MetS components was not different in subjects carrying different PPARγ2 or ACE ID genotypes (Table 3). When subjects free from drug treatments were analyzed separately, a lower prevalence of MetS emerged in individuals bearing the ACE II genotype (p: 0.018).

**Table 3:** Number of components (Metabolic score) and prevalence of Metabolic Syndrome among different PPARγ and ACE genotypes in all the subjects studied and in the untreated patients group

		All Subjects						
		
		Pro12Ala			ACE I/D			
			
		PP (334)	XA (30)	p	DD (137)	ID (172)	II (55)	P
**All** **(n 364)**	**Metabolic score (n)**	2.6 ± 1.2	2.4 ± 1.2	0.53^a^	2.7 ± 1.3	2.5 ± 1.2	2.4 ± 1.1	0.23^a^
	**Metabolic syndrome (%)**	53.3	53.4	0.57^b^	58.7	51.5	45.5	0.20^b^

		**PP (143)**	**XA (18)**		**DD (55)**	**ID (84)**	**II (22)**	
		
**Not treated** **(n 161)**	**Metabolic score (n)**	2.01 ± 1.2	2.11 ± 1.1	0.74	2.11 ± 1.2	2.10 ± 1.2	1.55 ± 1	0.09
	**Metabolic syndrome (%)**	33.6	38.9	0.42	***42.9***	***34.9***	***9.1***	**0.018^b^**

^a ^ANOVA. ^b ^Chi-square analysis

## Discussion

We evaluated the possible association between PPARγ pro12Ala and ACE I/D gene polymorphisms and MetS, fat-mass, and fat distribution. As previously reported [17,19,35], we found that carriers of the Ala allele had higher BMI. Although some studies [13-16] have shown Pro12Ala to be associated with lower BMI, our results coincide with meta-analysis conducted by Masud et al. [18] that found Ala allele association with higher BMI, specifically in subject with mean BMI > 27, similar to our population BMI. We also found a significant gene-gender interaction with BMI and fat-mass significantly higher in women but not men. This is in contrast with the data recently reported by Morini et al. [36], in a population with the same genetic background. We cannot exclude that the lack of association in men might be due to the limited number of males and the consequent lack of statistical power.

Despite having higher BMI, XA subjects showed similar waist, fasting glucose, and fasting insulin compared to PP subjects. Moreover XA individuals showed lower systolic blood pressure. It appears from our data that the increase in BMI associated with PPARγ2 Ala genotype was not associated with a worse metabolic profile. We speculate that Ala genotype might reduce central fat distribution thus protecting from metabolic complication associated with increased BMI. This would be in good agreement with a previous report in a Spanish population [37]. This hypothesis would be supported by the finding that the regression between BMI and waist had a lower slope in XA individuals; despite not being significant. This result suggests that, for a given BMI, subjects carrying the Ala allele might have less visceral fat. Moreover, multiple regression analysis showed that Pro12Ala polymorphism was an independent predictor of waist circumference.

Several human and animal studies support a central role of PPARγ in adipose tissue distribution [38,39]. Ala genotype is considered to reduce PPARγ transcriptional activity; this reduced activity has been considered pivotal in reducing BMI and, ultimately, in the increasing insulin sensitivity in subjects with Ala allele [15,22]. However, the relationship between PPARγ activity and insulin sensitivity is far from being linear, and does not necessarily pass through quantitative modification of fat mass. Mechanisms regulating energy balance and metabolic profile involve complex interactions between genetic, environmental and behavioral factors. Genotype may predispose to site-specific adiposity, culminating in a state of energy imbalance. PPARγ appears to be a key regulator of energy balance, with polymorphisms on the PPARγ gene linked to obesity and effects on body composition [40-42]. Humans harboring loss of function PPARγ mutation (P467L) show a pattern of lipodystrophy and are insulin resistant [39]. Qualitative adipose tissue modification and altered adipose tissue distribution are key features in determining metabolic abnormalities in these subjects. In this view, our data support the idea that the increased BMI found in subjects with Ala allele may not be detrimental, similarly to what happens in TZD treated patients showing metabolic improvements despite increased body weight. Indeed, XA individuals had higher BMI but lower systolic blood pressure and similar lipid profile compared to PP subjects.

We couldn't find any difference between subjects with different ACE I/D polymorphism. Studies published so far reported both an association with MetS/hypertension or lack of association [43,44]. This inconstancy might depend from ethnic background, type of population (diabetic or no diabetic subjects), and diagnostic criteria chosen to define MetS. The NCEP ATPIII criteria are operatively simple to use. Nevertheless, individuals can be classified as hypertensive or diabetic only on the basis of the active use of antidiabetic or antihypertensive drugs, including ACE inhibitors. These drugs might be a confounder when analyzing the possible effect of ACE gene polymorphism. When we selected only pharmacotherapy naives subjects, we found a significantly lower prevalence of MetS in subjects carrying the ACE II compared to DD genotype.

We also found a significant gene interaction between ACE I/D and PPARγ2 Pro12Ala polymorphism on BMI and fat-mass, with subjects XA/DD being the ones with higher values. This is not surprising as recent studies showed a role of ACE gene in regulating body weight; indeed, an ACE-/- knock out mouse had reduced fat-mass and improved insulin sensitivity [45]. Moreover, Eisenmann et al. recently reported that children harboring the DD polymorphism have an higher BMI compared to II carriers [46]. From a mechanistic point of view, ACE DD subjects have higher levels of plasma ACE, and therefore potentially higher level of Angiotensin II which has been identified as a trophic factor in the differentiation of preadipocytes to mature adipocytes [47].

### Limits of our study

We are aware of the limit of our study in term of sample size, especially when carrying out subgroups analysis and that we must be cautious about drawing firm conclusions before validating them in a different, larger cohort.

## Conclusions

In conclusion we found that, despite a lack of association between Pro12Ala and ACE I/D polymorphism and MetS diagnosis, subjects carrying a PPARγ2 Ala allele have higher BMI and fat-mass but do not have a worse metabolic profile, probably because a more favorable distribution of adipose tissue. Pro12Ala polymorphism effect on BMI is not constant across gender, with significant association only found in woman. It exists a gene interaction between the two common polymorphism Pro12Ala and ACE I/D on BMI and fat mass. Further studies are needed to assess the real contribution of the PPARγPro12Ala polymorphism in determining adipose tissue distribution.

## List of abbreviations

MetS: Metabolic Syndrome; PPARγ2: peroxisome proliferator-activated receptor-γ2; ACE: angiotensin Converting Enzyme; BMI: body mass index.

## Competing interests

The authors declare that they have no competing interests.

## Authors' contributions

Authors contribute as following: EDN, AP: concept and design of the study, interpretation of the data and preparation of the manuscript; CM, FDV, MLM: data collection, critical revision of the manuscript; CB, JMS, CB: biological samples management and technical support, critical revision of the manuscript; RF: critical revision of the manuscript; GZ: interpretation of the data, and critical revision of the manuscript. All authors made contribution to drafting the article or revising it critically for important intellectual content. All authors have seen and approved the final version to be submitted.
